# Sequence Type 631 Vibrio parahaemolyticus, an Emerging Foodborne Pathogen in North America

**DOI:** 10.1128/JCM.02162-16

**Published:** 2017-01-25

**Authors:** Feng Xu, Narjol Gonzalez-Escalona, Julie Haendiges, Robert A. Myers, Jana Ferguson, Tracy Stiles, Eric Hickey, Michael Moore, John Michael Hickey, Christopher Schillaci, Laurn Mank, Kristin DeRosia-Banick, Nicholas Matluk, Amy Robbins, Robert P. Sebra, Vaughn S. Cooper, Stephen H. Jones, Cheryl A. Whistler

**Affiliations:** a Northeast Center for Vibrio Disease and Ecology, University of New Hampshire, Durham, New Hampshire, USA; b Department of Molecular, Cellular, and Biomedical Sciences, University of New Hampshire, Durham, New Hampshire, USA; c Genetics Graduate Program, University of New Hampshire, Durham, New Hampshire, USA; d Center for Food Safety and Applied Nutrition, Food and Drug Administration, College Park, Maryland, USA; e Department of Health and Mental Hygiene, Baltimore, Maryland, USA; f Massachusetts Department of Public Health, Boston, Massachusetts, USA; g Massachusetts Division of Marine Fisheries, New Bedford, Massachusetts, USA; h Department of Public Health Laboratory, Rocky Hill, Connecticut, USA; i Department of Agriculture, Bureau of Aquaculture, State of Connecticut, Milford, Connecticut, USA; j Department of Health and Human Services, Augusta, Maine, USA; k Icahn Institute and Department of Genetics & Genomic Sciences, Icahn School of Medicine at Mount Sinai, New York, New York, USA; l Microbiology and Molecular Genetics, University of Pittsburgh School of Medicine, Pittsburgh, Pennsylvania, USA; m Department of Natural Resources and the Environment, University of New Hampshire, Durham, New Hampshire, USA; The Johns Hopkins University School of Medicine

**Keywords:** Vibrio parahaemolyticus, core genome multilocus sequence type analysis, emerging pathogen, genomics, molecular epidemiology

## LETTER

Vibrio parahaemolyticus is the leading seafood-transmitted bacterial pathogen worldwide. It causes gastroenteritis and, rarely, lethal septicemia. The estimated 45,000 annual cases of foodborne V. parahaemolyticus infections in the United States are concerning because their incidences are rising despite control measures, in part due to the impact of changing climate on pathogen abundance and distribution (1; https://www.cdc.gov/vibrio/). Although the pandemic complex of strains of sequence type 3 (ST3) (serotype O3:K6) has dominated infections worldwide (2), in the United States and Canada, the most prevalent clinical strains are of ST36 (O4:K12), which recently spread from the Pacific into the Atlantic (3–8).

Here we report that a new lineage of V. parahaemolyticus, identified as ST631, is rapidly emerging as the predominant pathogenic clade endemic to the Atlantic coast of North America (3, 4, 8). The first reported ST631 genome came from a clinical case that occurred in Louisiana in 2007 and was traced to oysters from Florida (8). In 2009, a second ST631 clinical isolate was reported in Prince Edward Island, Canada (O11:KUT) (4). From 2010 to 2015, the incidence of infections by strains of ST631 has increased, with 35 confirmed cases reported in four Atlantic coastal U.S. states (Table 1), where they are second only to ST36 strains in prevalence. Due to the self-limiting nature of infections and underreporting (9), ST631 infections may be more widespread.

**TABLE 1:** ST631 isolates with relevant information

Isolate	SRA or GenBank accession no.^*a*^	State of isolation^*b*^	Trace-back location^*b*^	Yr of isolation	Reporting country^*c*^	Source^*d*^	Geographic location^*e*^
VP2007-095	SRR869104	LA	FL	2007	USA	C	FL
09-4436	LRAJ01000000	PEI	PEI	2009	Canada	C	PEI
S487-4	LFZE01000000	NA	Canada	2013	Canada	E	PEI
MAVP-A	SRR4032168	MA	NA	2010	USA	C	
MAVP-E	SRR1952988	MA	MA	2010	USA	C	GOM
MAVP-P	SRR4032175	MA	NA	2010	USA	C	
MAVP-T	SRR4032176	MA	NA	2010	USA	C	
MAVP-L	SRR4032169	MA	MA	2011	USA	C	GOM
MAVP-Q	SRR4035056	MA	MA	2011	USA	C	GOM
MAVP-4	SRR4032177	MA	NA	2013	USA	C	
MAVP-30	SRR4032178	MA	NA	2013	USA	C	
MAVP-39	SRR4032179	MA	NA	2013	USA	C	
MAVP-56	SRR4032180	MA	PEI	2013	USA	C	PEI
MAVP-74	SRR4032181	MA	CT or PEI	2014	USA	C	LIS or PEI
MAVP-75	SRR4032182	MA	CT or MA	2014	USA	C	GOM or LIS
MAVP-78	SRR4032170	MA	MA	2014	USA	C	GOM
MAVP-90	SRR4032171	MA	CT	2015	USA	C	LIS
MAVP-94	SRR4032172	MA	MA	2015	USA	C	GOM
MAVP-109	SRR4032173	MA	MA	2015	USA	C	GOM
MAVP-112	SRR4032174	MA	MA	2015	USA	C	GOM
VP1	SRR4032354	MD	VA	2012	USA	C	MAC
VP8	SRR4032362	MD	NA	2012	USA	C	
VP9	SRR4032363	MD	NJ	2012	USA	C	MAC
VP31	SRR4032355	MD	NJ	2013	USA	C	MAC
VP35	SRR4032356	MD	NA	2013	USA	C	
VP41	SRR4032357	MD	NA	2013	USA	C	
VP44	SRR4032358	MD	NA	2013	USA	C	
VP45	SRR4032359	MD	CT or VA	2013	USA	C	LIS or MAC
VP47	SRR4032360	MD	NA	2013	USA	C	
VP55	SRR4032361	MD	NA	2014	USA	C	
PNUSAV000012	SRR4016797	MD	CT, MA, or ME	2015	USA	C	GOM or LIS
PNUSAV000015	SRR4016801	MD	CT, MA, NY, PEI, or VA	2015	USA	C	GOM, LIS, MAC, or PEI
PNUSAV00021	SRR4018053	MD	NA	2015	USA	C	
CTVP27C	SRR4090622	CT	CT or VA	2013	USA	C	LIS or MAC
CTVP31C	SRR4090623	CT	NA	2013	USA	C	
CTVP34C	SRR4090624	CT	NA	2013	USA	C	
MEVP-12	SRR4090625	ME	NA	2015	USA	C	
MEVP-14	SRR4090626	ME	NA	2015	USA	C	

a Massachusetts, Connecticut, and Maine isolates were sequenced using the Illumina HiSeq2500 sequencer at the Hubbard Center for Genomic Studies at the University of New Hampshire, whereas Maryland isolates were sequenced using the Illumina MiSeq sequencer at the Center for Food Safety and Applied Nutrition, Food and Drug Administration, Maryland, or at the Department of Health and Hygiene, Maryland.

b Where available, the U.S. state or Canadian location of isolation and infection is identified. For multisource traces, all possible sources are listed. CT, Connecticut; FL, Florida; LA, Louisiana; MA, Massachusetts; ME, Maine; NA, information was not available or was not determined; NJ, New Jersey; NY, New York; PEI, Prince Edward Island; VA, Virginia.

c The country which reported the isolate.

d C, clinical isolate; E, environmental isolate (specifically, from an oyster).

e The geographic locations of the sources corresponding to those identified in Fig. 1A. These include Florida (FL), the Gulf of Maine (GOM), Long Island Sound (LIS), the Mid-Atlantic Coast (MAC), and Prince Edward Island (PEI).

Genome comparisons were used to understand the potential relationships of ST631 strains, which share no recent ancestry with and differ substantially from ST36 and ST3 strains (>3,600 out of 3,909 shared genes contained variation). ST631 has a virulence gene profile similar to that of ST36 in that it harbors *tdh*, *trh*, and a type 3 secretion system (T3SS2) and is urease positive. We applied a core genome multilocus typing (cgMLST) scheme to draft genomes of 37 clinical isolates and 1 environmental isolate (Table 1) representing the geographic and time spans of infections. This analysis identified 132 single nucleotide polymorphisms (SNPs) in the population and confirmed that clinical ST631 isolates are clonal, with limited diversification (Fig. 1). Within the ST631 population, 97% of the core genes are identical, whereas less than 8% of the core genes are identical between ST631, ST36, and ST3 strains. Both maximum-likelihood phylogeny and minimum spanning tree analysis indicated a mixed population (Fig. 1A and B). Most isolates grouped within one clonal complex, with only a few divergent isolates (Fig. 1B). This population structure suggests that this pathogenic lineage recently evolved and that its distribution may have expanded along the North American Atlantic Coast (10).

**FIG 1:**
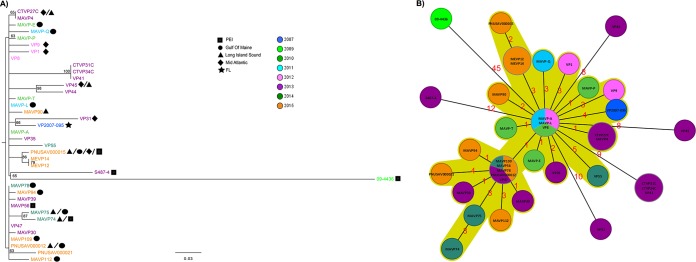
Phylogenetic relationships among ST631 isolates traced to the northwestern Atlantic (2007 to 2015). (A) A maximum-likelihood tree constructed with the core genome SNPs identified from cluster analysis (described below) of 35 newly sequenced clinical isolates reported in Massachusetts, Maryland, Maine, and Connecticut and 3 isolates whose draft genomes were publicly available in the NCBI database (strains 09-4436, S487-4, and VP2007-95) (Table 1) demonstrates the highly clonal nature of pathogenic ST631 isolates, which are colored by year and marked by geographic distribution. The scale bar represents the average number of nucleotide substitutions per site, and branches with greater than 60% bootstrap support are labeled. (B) A minimum spanning tree analysis reflecting the relationships among ST631 isolates based on core gene SNPs differences further demonstrates the clonal population structure. The numbers above the connected lines (not to scale) represent SNP differences. The isolates are colored by year of isolation using the same color scheme as in panel A. Cluster analysis of ST631 was performed using a custom cgMLST analysis using Ridom SeqSphere+ software v3.2.1 (Ridom GmbH, Münster, Germany). Briefly, the cgMLST software first defines a cgMLST scheme using the cgMLST target definer tool with default settings. MAVP-Q was used as the reference genome (4,568 genes). Then, five other V. parahaemolyticus genomes (strains BB22OP, CDC_K4557, FDA_R31, RIMD 2210633, and UCM-V493) were used for comparison with the reference genome to establish the core and accessory genome genes. Genes that are repeated in more than one copy in any of the six genomes were removed from the analysis. Subsequently, a task template that contains both core and accessory genes was created. Each individual gene locus from MAVP-Q was assigned allele number 1. Then each individual ST631 V. parahaemolyticus genome assembly was queried against the task template, during which any locus that differed from the reference genome or any other queried genome was assigned a new allele number. For the cgMLST, a gene-by-gene analysis of all core genes (excluding accessory genes) was performed and SNPs were identified within different alleles to establish genetic distance calculations. PEI, Prince Edward Island; FL, Florida.

The fact that an increasing number of cases tracing to sources in the northwestern Atlantic suggests that ST631 poses a mounting public health threat and calls for surveillance of this lineage to reduce illnesses. That its emergence coincided with warming ocean trends in some areas of the northwestern Atlantic (2) and invasion by a nonresident pathogen indicates that a changing climate may be driving pathogen dynamics (1, 2, 3, 7). However, this does not eliminate the potential of anthropogenic influences on the dissemination of ST631 strains, whose continued population expansion may increase human health risk beyond North America.

### Accession number(s).

Sequences were deposited in the Sequence Read Archive under accession numbers SRR1952988, SRR4016797, SRR4016801, SRR4018053, SRR4032168 to SRR4032182, SRR4032354 to SRR4032363, SRR4035056, and SRR4090622 to SRR4090626.
